# Functional characterization of retinal ganglion cells using tailored nonlinear modeling

**DOI:** 10.1038/s41598-019-45048-8

**Published:** 2019-06-18

**Authors:** Qing Shi, Pranjal Gupta, Alexandra K. Boukhvalova, Joshua H. Singer, Daniel A. Butts

**Affiliations:** 1 Department of Biology, University of Maryland, College Park, MD United States; 2 Program in Neuroscience and Cognitive Science, University of Maryland, College Park, MD United States

**Keywords:** Neural encoding, Computational neuroscience

## Abstract

The mammalian retina encodes the visual world in action potentials generated by 20–50 functionally and anatomically-distinct types of retinal ganglion cell (RGC). Individual RGC types receive synaptic input from distinct presynaptic circuits; therefore, their responsiveness to specific features in the visual scene arises from the information encoded in synaptic input and shaped by postsynaptic signal integration and spike generation. Unfortunately, there is a dearth of tools for characterizing the computations reflected in RGC spike output. Therefore, we developed a statistical model, the separable Nonlinear Input Model, to characterize the excitatory and suppressive components of RGC receptive fields. We recorded RGC responses to a correlated noise (“cloud”) stimulus in an *in vitro* preparation of mouse retina and found that our model accurately predicted RGC responses at high spatiotemporal resolution. It identified multiple receptive fields reflecting the main excitatory and suppressive components of the response of each neuron. Significantly, our model accurately identified ON-OFF cells and distinguished their distinct ON and OFF receptive fields, and it demonstrated a diversity of suppressive receptive fields in the RGC population. In total, our method offers a rich description of RGC computation and sets a foundation for relating it to retinal circuitry.

## Introduction

The early stages of sensory processing encode sensory input in a format that allows higher brain areas to extract information essential to guide behavior. In the retina, over twenty types of retinal output neurons – retinal ganglion cells (RGCs) – serve as “feature detectors” that each encode specific components of the visual world and convey them to the brain^1–3^. Feature detection is accomplished by distributing the output of photoreceptors to parallel circuits, each of which originates with one of ~15 types of bipolar cell and provides input to multiple types of RGCs^4^. Thus, responses of different RGCs reflect computations performed by the various presynaptic, parallel circuits^4^. Although the anatomy and retinal circuitry supporting the diversity of RGC types has been studied in molecular^5^, physiological^6^, and computational detail^7–9^, the methodology necessary to represent the diversity of retinal computations in RGC outputs is relatively anemic.

Being able to describe the “diversity of computation” requires sufficiently complex mathematical descriptions of how RGCs process visual stimuli. The most commonly used mathematical model of retinal computation is the linear-nonlinear (LN) model, which describes the input-output transformation of the circuit in two steps: one, a linear filter that emphasizes particular spatial and temporal features in the stimulus delivered to the photoreceptors; two, a nonlinear function that captures spike generation in RGCs^10^. The LN model is appealing in its simplicity and yields an easily interpretable spatiotemporal filter (also called the linear receptive field, or RF), which typically has a center-surround structure and either ON or OFF selectivity in time (i.e., its polarity indicates whether neurons respond to increases or decreases in luminance). The “spiking nonlinearity” describes how sensitively neuron firing rates depend on the stimulus^11^.

Although the LN model can differentiate ON from OFF cells based on their RFs, the single linear filter of the LN model condenses all retinal processing into a single step, which masks the diverse processing channels presynaptic to RGCs^12^. This is a particular problem in the mouse retina, where ON-OFF cells (i.e., cells that respond both to increments and decrements in luminance) are the most common type^13^. ON-OFF cells have at least one ON and one OFF RF, and a single linear RF therefore averages ON and OFF filters together^11,14,15^. Likewise, a linear filter cannot distinguish between ON excitation and OFF inhibition (or vice versa).

Thus, more sophisticated (and necessarily nonlinear) models that involve the characterization of multiple RFs are required to capture the diversity in retinal computation^15–18^. Nonlinear models are generally more difficult to fit to data because they require more parameters (e.g., multiple RFs) and a nonlinear description that can capture a sufficient range of computation. Here, we use a “space-time separable” form of the Nonlinear Input Model (NIM)^15^ to fit multiple excitatory and suppressive RFs by maximum-likelihood modeling. Space-time separability refers to representing spatiotemporal RFs as combinations of separate spatial and temporal filters; this permits much more efficient parameter estimation^15,18–20^. Furthermore, because this approach does not require uncorrelated “white noise” stimuli, we can characterize RGCs experimentally using tailored “cloud” stimuli, which have spatial features on multiple scales. The NIM described detailed spatiotemporal RF maps in ON, OFF, and (uniquely) ON-OFF RGCs in the mammalian retina, and it revealed suppressive RFs unobserved in standard LN analyses. Such detail provides a much fuller picture of the computations represented in RGC outputs and provides the means to understand their functional diversity.

## Methods

### Neurophysiology

Experimental use of mice was performed under an animal protocol approved by the IACUC of the University of Maryland. All methods were performed in compliance with National Institutes of Health guidelines and regulations. C57bl/6 mice of either sex were dark-adapted for 1–2 hrs before isoflurane anesthesia followed by euthanasia by decapitation. Eyes were removed and retinas dissected free in Ames’ medium (Sigma) bubbled with 95% O_2_/5% CO_2_ (Carbogen) at room temperature. Ventral retinae were isolated in small, rectangular sections and placed RGCs down on a 6 × 10 perforated multielectrode array (Multichannel Systems, Tübingen, Germany). After a rest period of at least 30 minutes (to permit tissue adhesion to the MEA), RGC responses to light stimuli were recorded while the tissue was perfused with Ames’ medium bubbled with Carbogen and kept at 32 °C. All dissection procedures were performed under dim red illumination.

### Stimuli

**Figure 1:**
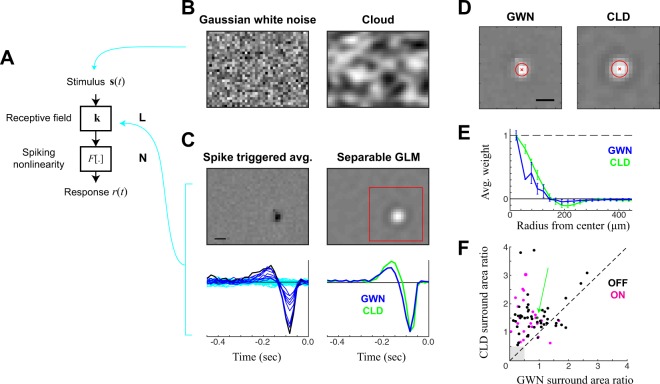
Estimation of spatiotemporal RFs using separable GLM and cloud stimuli. **(A)** Schematic for the Linear-Nonlinear (LN) model of ganglion cells: consisting of a linear spatiotemporal filter k, followed by a spiking nonlinearity F[.]. **(B)** Example frames from the two stimulus classes used for model estimation: gaussian white noise (GWN, left) and cloud stimuli (CLD, right), with the CLD stimulus defined as a spatially low-pass filtered version of the GWN. (**C-E**) Analysis of an example OFF cell presented with both GWN and CLD stimuli. (**C)** LN model filters, fit using spike-triggered averaging (STA) applied to GWN data (left), compared with a space-time separable generalized linear model (GLM) fit to the cloud stimulus (right). The STA yields a temporal function for every pixel (bottom left, with traces colored by their overall amplitude), from which the spatial map at the best latency can be extracted (top left). By comparison, the separable GLM extracts a single temporal function (bottom right), which is multiplicatively combined with a single spatial function (top right). This reveals a much less noisy spatial estimate, from which an opposite-sign surround is visible. The temporal kernel is also shown for the separable GLM fit to the GWN stimulus, revealing a slightly longer-latency response, but otherwise very similar temporal tuning. (**D)** Spatial maps derived from a separable GLM applied to the GWN (left) and CLD (right) stimuli, revealing the ability of the CLD stimuli to drive clearer spatial information at larger scales: ‘x’ shows the estimated center, and one standard deviation shown by the red circle. (**E)** To measure the presence of the spatial surround, the average pixel intensity (and standard deviation) at each radius is plotted versus the radius. This also demonstrates the ability of the CLD stimulus (green) to more robustly recover the surround. **(F)** The surround-area-to-center-area ratio for CLD vs. GWN stimuli, shown for each ON and OFF cell where both conditions were recorded (N = 81, where 7 cells with negligible surround areas (gray square) not shown). The green arrow marks the example cell shown in C–E. Scale bars are 200 μm.

Visual stimuli were generated using the *Psychophysics Toolbox* in *Matlab* and presented in the UV spectrum using a modified DLP projector (*HP Notebook Projection Companion Projector*, Model: HSTNN-FP01) (frame rate = 60 Hz). Projector output (*I*_mean_ ≈ 5 × 10^3^ photons/cm^2^/s, 398 nm; measured using a fiber-coupled spectrophotometer: Ocean Optics USB4000, Ocean Optics, Dunedin, FL, USA) was focused through the MEA and onto the photoreceptor layer using a microscope objective (Olympus) optimized for UV transmission. The mouse retina contains both S and M opsin- expressing cones found in a ventral→dorsal gradient, with S-opsin dominating phototransduction signaling in the ventral retina^21–23^. Here, we chose to use a UV stimulus because the mouse retina responds across a broader frequency range in response to UV as opposed to green stimuli^24^. Stimuli include: (1) 120 repeats of a whole-field stimulus that alternated between black and white, changing every 500 ms; (2) Gaussian white noise (GWN) flickering checkerboard (pixel size = 44.77 µm); (3) spatially correlated “cloud” stimulus; and (4) repeated, short periods of cloud (short repeats) for cross validation. The cloud stimulus was generated by low-pass filtering the GWN using a two-dimensional spatial Gaussian filter centered at the origin (in Fourier space) with a standard deviation of ~1.3 cycles/mm. Note that while this does not result in any single spatial scale, it does give the appearance of features roughly on the order of RF field sizes, as demonstrated in Fig. 1. The total duration of GWN, cloud, and short repeats was 20 min, and was broken into two 10-min blocks, separated by 10-min resting intervals. A 60-s long conditioning, whole-field gray light at *I*_mean_ was presented at the beginning of each stimulus (block). Data was recorded at 50 kHz using the MC_Rack software (Multichannel Systems, Tübingen, Germany). Spikes were sorted using an offline sorter (Plexon Inc).

### Data analysis

A majority of experiments and neurons were recorded using just cloud stimulation (24 preparations, 431 well-isolated RGCs). Due to the instability of long recordings over the two 10-min blocks of cloud stimulus and repeats, we only used both blocks to fit models for 12 of the 24 experiments, using two criteria: (1) average firing rates of neurons did not change more than 50% from the first to second experiment; (2) cross-validated likelihood of the LN model did not change by more than 50%. If neither condition was met, we used the block which contained (on average) the best fits without suppressive terms added (LN or ON-OFF fits). We also recorded responses to a novel 10-sec repeated stimulus sequence, although only a handful of these were stable stable by these criteria. As a result, we were able to generate PSTHs to compare to model predictions in the cases shown in Figures, but did not use these numbers in population statistics.

For a smaller set of experiments (6 experiments, 125 well-isolated neurons), we recorded responses to both cloud and GWN stimuli in order to facilitate comparisons of spatial RFs between conditions. Here, 81/125 neurons with clear temporal kernels in the sGLM fits for both cloud and GWN conditions and RFs did not overlap with the edge of stimulation were selected for analysis. Note that these comparisons were based on comparing ON an OFF RFs (without regard to later analyses identifying ON-OFF neurons), and thus would implicitly eliminate some ON-OFF cells with strange linear RFs. Also, because performance comparisons were not important for this analysis, we did not use the stringent stability above for rejecting segments of the data.

Data were binned at exactly twice the stimulus refresh frequency (roughly 16 ms) for all subsequent analyses.

### Linear and Nonlinear modeling

This work follows general modeling for the Nonlinear Input Model (NIM) described in detail in McFarland *et al*.^15^; modified to incorporate separable spatiotemporal RFs. As described below, the model fitting code (in *Matlab*) has been made publicly available, and should complement the descriptions here and in previous publications. Briefly, all models were fit to maximize the penalized Poisson log-likelihood of the model given the data^25^, given by:

$$LL=\sum _{t}[{R}_{obs}(t)\mathrm{log}\,r(t)-r(t)]-P[\theta ]$$

where *r*(*t*) is the predicted spike output of the model, *R*_*obs*_(*t*) is the binned spike count of the neuron being modeled, and *P*[*θ*] is a regularization penalty that depends on the model parameters *θ*.

The predicted firing rate of the GLM model is given, familiarly, by *r*(*t*) = *F*[**K**∙**s**(*t*)]^25,26^ (also Eq. 1, below), where we are using vector notation [**bold**] to denote the linear projection of the stimulus at a given time point (which includes all spatial positions and time lags) and the spatiotemporal filter **K** = *K*(*x*, *y*, *τ*) over all spatial positions and lags (back in time). The spiking nonlinearity *F*[.] is defined to be *F*[*x*] = log[1 + exp(*x*)]^15,25^. For the separable GLM, we approximated the full spatiotemporal kernel *K*(*x*, *y*, *τ*) as the outer product of a spatial kernel *k*_*sp*_(*x*, *y*) and temporal kernel *k*_*t*_(*τ*), resulting in the separable GLM (sGLM) used below.

The predicted firing rate of the NIM is given by^15^ (Eq. 2, below). As with the sGLM, the separable NIM (sNIM) involved approximating each subunit RF as the outer product of a spatial and a temporal kernel.

Because the outer product does not uniquely specify either kernel (because one can multiply one by any constant and divide the other to get a mathematically equivalent result), we constrained the spatial kernels to be normalized (i.e., fixed overall magnitude), and to have positive centers.

Models were fit using gradient ascent of the *LL* (above), as described in detail previously^15^. However, due to the separable kernels, each RF was fit by alternating fits of the spatial component (holding the temporal component fixed) and temporal component (holding the spatial component fixed) until convergence. For each neuron, we first fit computed the spike triggered average, and then used singular value decomposition to initialize the sGLM. Once the sGLM was fit, we initialized an sNIM to have ON-OFF selectivity: with two excitatory kernels, where the first kernel was initialized with that of the sGLM, and the second had the same temporal kernel multiplied by −1. If the result had a better *LL* than the LN fit and maintained an ON-OFF form, we then fit models with additional suppressive terms. Otherwise, we generated an NIM with a single excitatory subunit (initialized using the sGLM) and added suppressive terms. Note that each subunit could be one of four general types given the range of kernels that were fit: 1) ON excitatory, 2) suppressive, 3) OFF excitatory, and 4) OFF suppressive. ON and OFF refer to the preferred stimulus, and excitatory and suppressive indicate whether the filter output will be positive or negative.

All models were regularized using a Laplacian square penalty separately on the spatial and temporal kernels – which penalizes the second derivative^15^, resulting the penalized log-likelihood (above).

### Spatial RF analysis

The robust identification of multiple spatiotemporal RFs contributing to a given RGC response allows for detailed analysis of their spatial integration properties, as well as detecting the presence of surrounds. To this end, we fit circular Gaussians to a given spatial RF, fitting the precise center, standard deviation, and amplitude (4-parameters) by minimizing the mean-squared error using simplex optimization. We also could fit elliptical Gaussians (two additional parameters) robustly, but we found that this did not reveal additional useful information. With the centers identified, we could then take the average magnitude of pixels at any given radius from the center, generating a radial profile *w*(*r*) that revealed the center and surround. We also reported the center and surround areas, given by identifying the zero-crossing of *w*(*r*) and performing a weighted integration of (2π*r*)*w*(*r*) on either side of this zero-crossing.

### Availability of modeling code

All modeling code is available through our lab website at http://neurotheory.umd.edu/Code.

## Results

We recorded retinal ganglion cells (RGCs) from *in vitro* mouse retina using a multielectrode array. We first consider recordings made during the presentation of spatiotemporal white noise in order to perform standard receptive field (RF) analyses. The RF of RGCs is usually defined to be the optimal linear filter k that best predicts the cell’s spike responses^27^ and is the basis of the Linear-Nonlinear (LN) model, which generates a firing rate prediction of the response *r*(*t*) given the stimulus **s**(t) as follows (Fig. 1A):

1 $$r(t)=F[{\sum }_{i}{k}_{i}{s}_{i}(t)]\equiv F[{\bf{k}}\cdot {\bf{s}}(t)]$$

where *i* indexes the components of **s**(*t*) and the corresponding filter **k** that are relevant to the response at time *t* (including spatial locations and time lags into the past), and *F*[.] is a spiking nonlinearity that maps the output of the filter to a firing rate. Note we are using boldface to represent vectors, i.e., **k** = [*k*_1_ *k*_2_ *k*_3_ …]. When the stimulus is uncorrelated across its dimensions, then the optimal linear filter can be simply estimated using the spike triggered average (STA)^10,27^, defined as the average stimulus that evoked a spike. The spiking nonlinearity also can be estimated easily using the histogram^10,27^. For this reason, the LN model estimated in the context of Gaussian white noise (GWN; Fig. 1B, *left*) is the predominant method used to characterize RGCs^10^.

In using a white noise stimulus, however, consideration of the spatial extent and temporal duration of the pixels that compose the stimulus is critical^28,29^. Spatial and temporal parameters often are chosen empirically either to drive strong RGC responses or to yield RFs with good appearances, or both. A good RF for RGCs usually has a relatively smooth center covered by a few pixels (Fig. 1C, *left*); note, though, that in many publications the RFs pictured are interpolated into a higher resolution image and smoothed. While, in principle, smoother RFs can be obtained with finer spatial resolution using smaller pixels, having a large number of uncorrelated pixels tiling any one feature (e.g., the RF center) will typically not drive robust responses, because the summed luminance over that area will not deviate much from gray (mean) luminance. In the context of MEA recording, where RFs of many sizes might be sampled, the stimulus is usually adjusted to the most prevalent RF sizes, and therefore can be maladjusted for others^29^.

Furthermore, even an optimal choice of pixel size for a given RF center can bias RF estimation to features of that size. Receptive fields that have larger scales, such as the surround, will not be driven effectively by GWN stimuli optimized for the center: again, this occurs because the uncorrelated light and dark pixels will average to gray and not drive the larger spatial features. Consequently, features such as the RF surround will contribute minimally to the response to GWN stimulation and often not be visible in the spike-triggered average (Fig. 1C, *left*), which is a common issue with GWN characterizations throughout previous work.

### Likelihood based estimation of RFs using cloud stimuli

We presented a “cloud” stimulus to the same neurons characterized using GWN. This cloud stimulus was designed to have the same pixel size and duration as the GWN stimulus, but it introduced spatial correlations within each frame; these were generated by applying a spatial low-pass filter to a GWN stimulus (see Methods). Such correlations implicitly lead to dark and bright areas at a range of spatial scales (Fig. 1B, *right*) while maintaining the same high spatial resolution as GWN. The introduction of such correlations confounds traditional spike-triggered characterizations because the presence of correlations requires an additional (and noisy) step in computing the optimal linear filter: deconvolution of stimulus correlations from the STA^27,30^. This problem can be avoided by direct estimation of the linear filer by maximum a posteriori estimation, such as in the framework of the Generalized Linear Model (GLM)^25,26^. Such optimization is easily performed in principle (given standard computers and software), but it can introduce problems of overfitting due to the large number of parameters in the spatiotemporal filter that must be estimated simultaneously. For example, an appropriate spatial and temporal resolution for our experiments (21 × 21 spatial grid and 40 time lags at 16 ms resolution) results in thousands of parameters (17,640 in this case) that must be fit simultaneously. Application of smoothness regularization can mitigate overfitting, but its ability to suppress the noise in the RF without over-smoothing is limited, given typical amounts of data. Thus, in the context of relatively high-resolution spatiotemporal estimation problems, the GLM often provides little (if any) advantage over the STA in the context of a GWN stimulus; STA estimation is not affected by the number of parameters because each element is an independently performed average.

This major limitation of GLM estimation of cell RFs can be overcome by low-rank approximation^18–20^, which involves representing the spatiotemporal RF as a combination of separately estimated spatial and temporal RFs. Therefore, we adapted GLM estimation techniques to estimate space-time separable filters by representing the spatiotemporal filter as a spatial filter multiplied by a temporal filter, i.e., *k*(*x*, *y*, *τ*) = *k*_*sp*_(*x*, *y*) × *k*_t_(*τ*). This separable form is fit through block-coordinate descent: after initializing with the [correlated] STA, the spatial filter is held fixed while the temporal kernel is estimated (40 parameters), and then the temporal kernel is held fixed while estimating the spatial kernel (441 parameters). Alternating continues until the fit converges (see Methods), and corresponding spatial and temporal regularization is applied to each separately. Such an approach yields clean estimates of both (Fig. 1C, *right*): with much smaller amount of noise than the STA, and the appearance of additional details of the RF such as an opposite-polarity surround.

The separable GLM can be applied to both GWN and cloud data, yielding nearly identical temporal kernels (Fig. 1C). RFs measured from the cloud stimulus context, however, yield much more consistent features (Fig. 1D). Note that both RFs are optimized in their respective contexts: differences in spatial structure represent the spatial patterns that best predicted responses using an LN model. Although the neuron shown fired similar number of spikes in each context (58,219 in GWN and 61,358 in CLD), RF elements of the cloud drive the RGC much more efficiently, leading to more robust features in the estimated RF (Fig. 1D) including a smaller latency to spike (Fig. 1C, *bottom right*). This is most evident in the appearance of the RF surround, which we measured by fitting the center to a circular Gaussian (Fig. 1D, red) and then measuring the average value of the RF at concentric distances to yield a precise measurement of its strength (Fig. 1E). While there was a large diversity in surround strength for the LN models measured across neurons, the cloud stimulus yielded stronger surrounds in all but a handful of cases (Fig. 1F).

### Estimation of ON-OFF receptive fields

**Figure 2:**
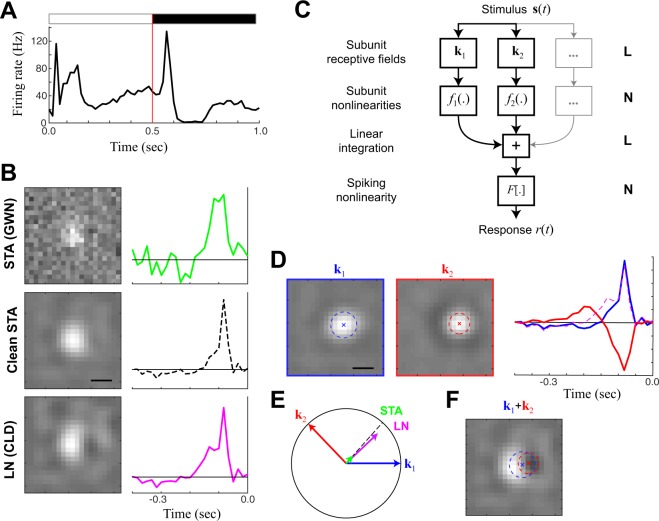
Receptive field mapping of ON-OFF cells. **(A)** The PSTH of an example ON-OFF cell in response to a full-field flashed stimulus alternating between black and white at 2 Hz. **(B)** The LN model (estimated using the STA and separable GLM) are limited to compute a single receptive field for the cell. As with Figure 1, the separable GLM (bottom) reveals the most spatial detail relative to the STA in GWN (top), although can be ‘cleaned’ (middle, see E) to reveal a similar receptive field as the GLM. **(C)** The structure of the NIM model, which is an LN-LN cascade that can identify several features (each with its own receptive field) that nonlinearly combine to result in the spike output. **(D)** The separable NIM (sNIM) finds two excitatory receptive fields for the ON-OFF cell. By convention, the spatial map (left) is always positive, and the temporal kernels (right) demonstrate that one is ON and the other OFF. For comparison, the LN filter from B is shown as a dashed magenta line. **(E)** The ON and OFF spatiotemporal filters of the sNIM can be considered vectors in high- dimensional stimulus space, and define a plane, with the circle showing unit-length in the plane that denotes whether a given filter fully projects into the plane. The LN model filter (magenta, from B) projects largely into this plane at the average position between the two sNIM vectors. Because of noise, the STA (green, also from B) largely projects outside of the plane, but its projection into the plane matches the LN filter, and can be “cleaned” by normalizing this in-plane projection. **(F)** As suggested by (E), simply averaging the ON and OFF NIM filters together produces a spatial map very similar to that of the LN model, with almost unnoticeable dark patch a result of the spatial offset of the OFF receptive field relative to the ON receptive field. Scale bars are 200 μm.

A large fraction of mouse RGCs are ON-OFF, meaning they are excited by both increments and decrements in luminance (Fig. 2A). Such selectivity defies characterization with linear RFs: linear stimulus processing implicitly averages the selectivity of different RF components into a single RF, and overlapping ON and OFF components cancel, i.e., (**k**_on_ · **s**) + (**k**_off_ · **s**) = (**k**_on_ + **k**_off_) · **s**. For two RF components to contribute separately to excitation, ON and OFF selectivity must be represented in an additional nonlinear stage of processing (Fig. 2A):

2 $$r(t)=F[{f}_{1}[{{\bf{k}}}_{1}\cdot {\bf{s}}(t)]+{f}_{2}[{{\bf{k}}}_{2}\cdot {\bf{s}}(t)]]$$

This model has the form of an LNLN cascade^31–33^ and has been formulated in a maximum likelihood context as the Nonlinear Input Model (NIM)^15^. We adapted the NIM to incorporate separable spatiotemporal filters, and the resulting model, the *separable NIM* (sNIM) was fit to the same data as the separable GLM described above. To begin, we show an example cell that responds to both increments and decrements in luminance (i.e., ON and OFF stimuli) in a slowly modulated full-field stimulus (Fig. 2B). The selectivity demonstrated by the STA suggested that this cell is an ON cell because the calculation is limited to computing a single RF. The separable GLM, however, provided additional resolution and revealed a spatial asymmetry in the spatial RF (*right*), suggesting spatially offset and partially cancelling ON and OFF components. The sNIM fit to the same data demonstrated robust, circular ON and OFF responses, each with a discernible surround (Fig. 2C). The resulting two filters are represented as vectors that define a plane (Fig. 2E) in the much higher-dimensional “stimulus space”, which represents all possible types of feature selectivity. This plane defined by the detected ON and OFF filters largely contains the GLM filter, which in this case matched the sum of the two NIM filters (Fig. 2F). Although most of the power in the STA is dominated by noise, its projection into this plane is also in the same direction as the GLM (Fig. 2B, *middle*).

**Figure 3:**
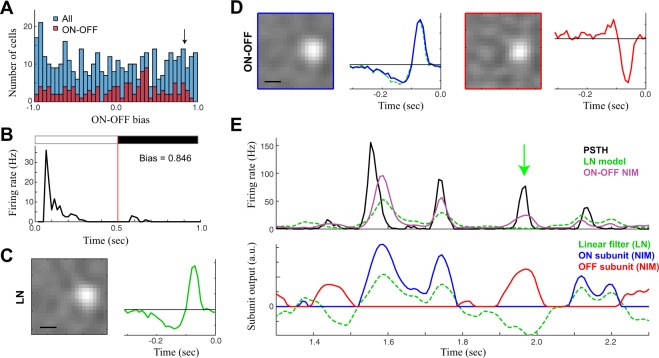
Hidden ON-OFF cells. **(A)** ON-OFF cells are often distinguished using their response to full-field flashes (as in Fig. 2A), leading to a simple measure of ON-OFF bias that is simply the normalized difference in response magnitude between ON and OFF flashes. This leads to a continuum of values across all ganglion cells (blue, all cells). However, using the sNIM to discriminate ON-OFF cells based on the presence of excitatory ON and OFF subunits, yields a similar continuum of values for ON-OFF bias (red). **(B)** The PSTH in response to full-field flashes of an example ON-OFF cell identified by the sNIM, but has a high ON-OFF bias consistent with an ON cell. **(C)** The same example cell’s linear receptive field is also consistent with an ON classification. **(D)** However, the sNIM pulls and ON and OFF excitatory receptive field. The ON receptive field nearly matches that of the LN model (green). **(E)** To demonstrate it is indeed an ON-OFF cell, the response to repeated presentations of the CLD stimulus is shown (top), relative to predictions of the ON-OFF sNIM (purple) and LN model (green). The underlying output of each subunit is shown below. The green arrow denotes an example of a response to an OFF stimulus, demonstrating it indeed has excitatory OFF responses. Scale bars are 200 μm.

The sensitivity of the sNIM reveals ON and OFF selectivity in RGCs that otherwise would appear to be exclusively ON or OFF cells. ON-OFF cells are often characterized based on response to increments and decrements in full-field luminance (e.g., Fig. 2A). Measures of the peak responses to ON and OFF steps^7^ yield a distribution of response types (Fig. 3A, blue), ranging from pure ON to pure OFF with a large number of intermediate values. Such measures, however, are poor indicators of true ON-OFF cells, as demonstrated by the histogram of values for neurons with clear ON and OFF excitatory components in the sNIM (red). For example, we show an example neuron having a negligible response to a full-field decrement in luminance (Fig. 3B) and a clear ON RF predicted by the GLM (Fig. 3C). This cell, however, is best described by an ON-OFF sNIM (Fig. 3D), and its ON-OFF selectivity can be demonstrated from analysis of responses to repeated presentations of a unique cloud stimulus (Fig. 3G).

**Figure 4:**
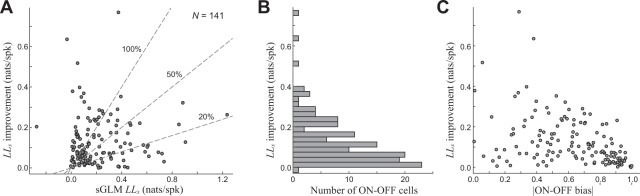
Cross-validated performance of ON-OFF sNIM. **(A)** The cross-validated log-likelihood (LLx) improvement of the ON-OFF sNIM with two excitatory RFs (e.g., Figs. 2, 3), relative to the LLx of the separable GLM. **(B)** A histogram of these LLx-improvements demonstrate most ON-OFF sNIM models contribute little to the model performance. **(C)** However, this can be largely explained by the distribution of ON-OFF bias (absolute value shown on horizontal axis), such that ON-OFF cells that are dominated by ON or OFF component (i.e. not balanced), will tend to show smaller improvements.

We thus characterized neurons as being ON-OFF based on whether their best model (i.e., that with the highest cross-validated model performance) had two excitatory subunits with opposing polarity. We used the log-likelihood (cross-validated, *LL*_*x*_), which is ideal for long continuous trials because it does not require repeated stimuli to compute (as compared with the more common measure such as explainable variance R^2^), as a measure of model performance^33,34^. The *LL*_*x*_-improvement for the ON-OFF model relative to the separable GLM was quite variable across labeled ON-OFF cells (Fig. 4A), with a median improvement of 55% (*N* = 141). There, however, were a large number of ON-OFF cells with small improvements (Fig. 4B). However, this was largely due to the distribution of ON-OFF bias (Fig. 4C), such neurons that were highly dominated by ON- or OFF- responses had little to gain by correctly predicting the response to opposite polarity, and thus large ON-OFF bias was associated with very little model improvement. While the PSTH-based analysis shown above (Fig. 3G) can demonstrate the clear presence of ON and OFF responses, such analysis was not possible with most of the dataset, because it depended on having repeated stimuli for each neuron that happens to contain noise patterns matching its ON and OFF RFs. As a result, model-based labeling cannot be definitive, but rather demonstrates the much more wide-spread presence of ON-OFF selectivity than what is traditionally reported through electrophysiological characterizations.

### Detection of suppressive surrounds

**Figure 5:**
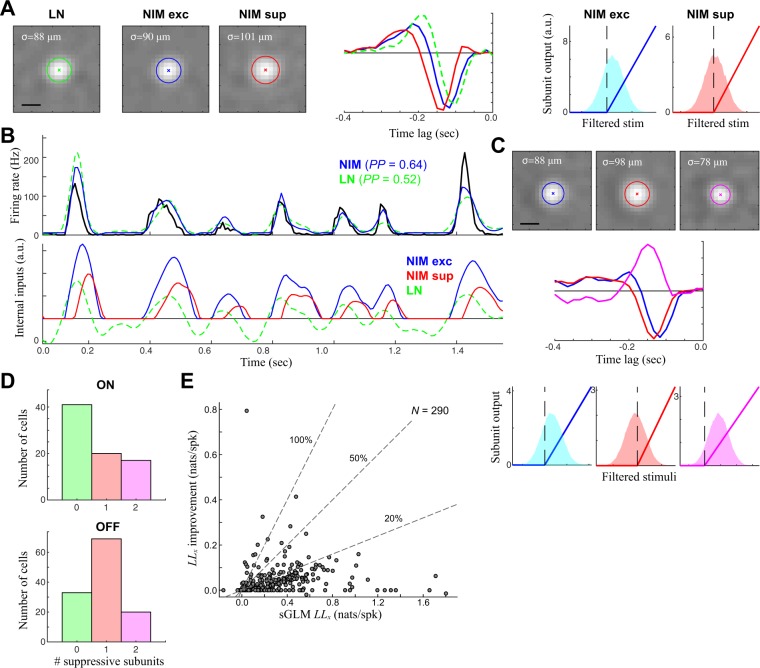
Detection of nonlinear suppression in a majority ganglion cells. **(A)** The sNIM model components for a typical OFF cell, which is best modeled by an excitatory (blue) and delayed suppressive (red) subunit. Both spatial components (left) are slightly larger than that predicted by the LN model, delayed in time relative to the LN model (middle), and strongly rectified (right). **(B)** The resulting sNIM predicts the response better (top), although the underlying excitation and delayed suppression largely cancel (bottom) in order to offer only subtle differences between the LN model and sNIM. **(C)** A different example OFF cell with two suppressive subunits: a delayed same- sign suppression (as with the example in A), as well as an opposite-sign ON suppression. **(D)** The distribution of detected subunits for ON (up) and OFF cells (down), demonstrating that a majority of ON and OFF cells have underlying suppressive receptive fields in addition to standard excitatory subunits that largely confer their tuning. **(E)** The cross-validated log-likelihood (LLx) improvement of adding suppression across all ON and OFF cells, relative to the LLx of the separable GLM.

Although ON-OFF cells are the most obvious examples of nonlinear processing in the retina, most RGCs exhibit some form of nonlinear processing in their responses, such as adaptation to stimulus contrast^35–38^ and the generation of temporal precision^39–41^. In the context of the NIM model, such nonlinear properties might be explained through the addition of suppressive subunits^33,42–45^, e.g., the same model structure that fit ON-OFF cells (Fig. 2C) with the second subunit’s output multiplied by −1 such that it can only suppress the firing rate. Indeed, the majority of OFF cells have suppressive RF(s) identified by the sNIM (e.g., Fig. 5). These suppressive subunits typically have roughly the same spatial extent as the excitatory subunit (and LN model RF) (Fig. 5A, *left*), but delayed in temporal kernel (Fig. 5A, *middle*). Such delayed suppression is consistent with similar models fit to cat RGCs^44^ and LGN cells^15,33,44^. Compared with the corresponding LN model, both the spatial and temporal filters of the NIM have broader dimensions; likely because the LN model’s RF is the average of filters that largely cancel each other. The contribution of the suppression tends to make the response more transient due to the delay^33^.

Other RGCs models had an opposite-sign (or “Pull”) suppression in addition to a same-sign suppression (Fig. 5C). There was in fact every combination evident in the sNIM fits to ON and OFF cells, with a majority of OFF cells having a detectable suppressive component, while slightly more than half of ON cells did not (Fig. 5D). However, the detection of suppressive RFs typically lead to much smaller improvements in model performance (Fig. 5E; median = 10.0%, *N* = 290), because the effect of suppressive RFs only had a small impact on the predicted firing rate (Fig. 5B), related to its precision^33^. Nevertheless, the detection of the diversity in suppressive RFs demonstrates the ability to more accurately reflect the diverse computation of different RF types.

**Figure 6:**
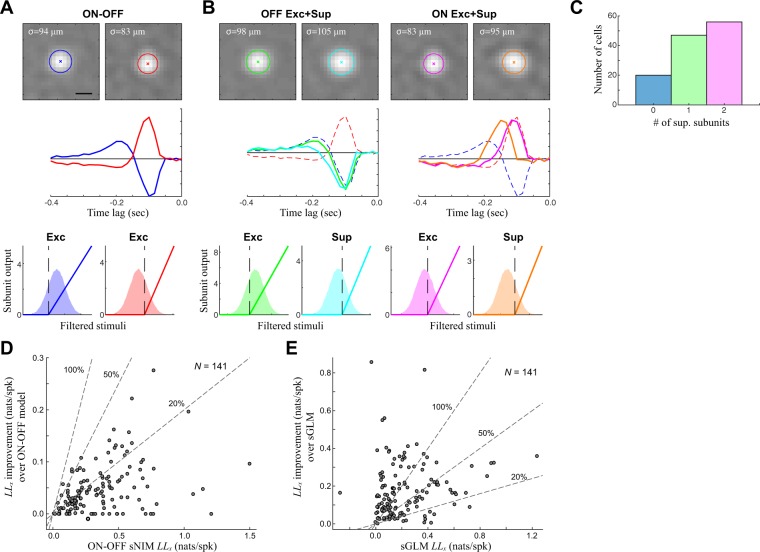
ON-OFF cells with delayed suppressive subunits. **(A)** A typical ON-OFF model with two excitatory subunits can also be better modeled by the addition of suppressive subunits. **(B)** A four-subunit model of the same ON-OFF cell, demonstrating a similar pattern of delayed suppression as the ON and OFF cells (Fig. 5): with each excitatory subunit having a paired, delayed, suppressive subunit. **(C)** A majority of ON-OFF cells also had detectable suppressive subunits. **(D)** The cross-validated log-likelihood (LLx) improvement of adding suppression across ON- OFF cells, relative to the LLx of the ON-OFF sNIM considered in Figs. 3, 4. **(E)** The LLx-improvement of the full sNIM relative to the sGLM across ON-OFF cells.

Suppressive RFs were also detected by the sNIM in ON-OFF neurons. Indeed, for the example neuron described by an ON and OFF excitatory RF (Fig. 6A), a delayed suppressive RF could be added to each excitatory RF (Fig. 6B). This pattern held in a majority of ON-OFF cells (Fig. 6C), although – like with the ON and OFF cells considered earlier, the addition of suppression only led to small improvements in model performance over the already large improvements associated with the ON-OFF models without suppression (Fig. 6D; median = 14.0%). However, when the sNIM is considered as a whole, it does result in considerable model improvements relative to the separable GLM (Fig. 6E; median = 89.7%, *N* = 141). Thus, the diversity of suppression is another element of the sNIM that contributes to the computational diversity of RGCs.

## Discussion

Here, we present a state-of-the-art modeling approach for characterizing retinal computation. It is based on extracellular recording of RGC action potentials evoked by “cloud” stimuli, which are correlated noise^46,47^. Analysis of responses to clouds reveals selectivity of RGCs across spatial scales: here, we used a maximum likelihood estimation applied to an LNLN cascade, which is selective to multiple spatiotemporal “features” (i.e., receptive fields), to describe stimulus-driven spike train data. To handle the large number of spatial and temporal dimensions of RFs, we adapted fitting procedures of the Nonlinear Input Model^15^ to use space-time separable RFs (i.e., the separable NIM; sNIM). Using this experimental and computational approach, we identified a diverse array of computations in the observed RGC population, which included a large number of ON-OFF cells. Our approach to characterizing RGC receptive fields (RFs) can be extended to detail differences between individual RGC types as well as between the retinas of different species (primate, mouse, salamander, etc.).

The sNIM utilizes a range of methods from existing statistical modeling methods; combining them to generate a robust description of retinal computation is novel and unique. The sNIM is based on the methods of the NIM^15,33,44^, modified to have space-time separable RFs^15,18–20,33,48^ to make it feasible to fit multiple spatiotemporal RFs per neuron at high spatial and temporal resolution. Furthermore, we employ cloud stimuli^46,47^ – essentially white noise stimuli filtered to have spatial correlations – to drive responses to features across spatial scales. This facilitated robust fitting of salient features of RGC computation, including separate ON and OFF and suppressive RFs.

Statistical models of RGCs (and related LGN neurons) are some of the most successful in neuroscience, in large part due to their predominantly linear behavior^49^. As a result, models of RGCs have been largely based on spike-triggered averages (STAs)^10^, which – although not explicitly “fit” to maximize likelihood – are the optimal linear filters in the context of stimuli such as Gaussian white noise^10,27^. Such models, however, capture only linear stimulus processing (i.e., a single RF) and must be used with uncorrelated stimuli. Spike-triggered covariance analysis^50–52^ extended spike-triggered techniques to nonlinear models, although are only able to yield a “feature space” (e.g., Fig. 2), within which the true features that the neuron is driven by exist. Thus, while such approaches identify multiple excitatory and suppressive features, they cannot be used to characterize these separate components contributing to RGC processing^14,15,33,52^. Furthermore, STC-based approaches^14,17,50^ can require a prohibitive amount of data for the types of spatiotemporal characterization, and for example could not be applied for recordings considered here.

The use of rectified nonlinearities in the context of an LNLN cascade, however, more clearly distinguishes the separate features that contributing to RGC computations, due to the two-stage nonlinear processing in such cascades better approximating the form of nonlinear processing in the retinal circuit^15,18,44^. Such an approach was recently used in the salamander retina, where it identified multiple excitatory features comprising single OFF RFs, likely corresponding to separate bipolar cell inputs^18^. This detection of excitatory RFs in another recent study in salamander was able to picture the spatial arrangement of such putative bipolar cell inputs in two-dimensions^17^. Neither study, however, detected ON-OFF cells nor suppressive RFs, likely reflecting the predominance of OFF RGCs in the salamander retina^9^. These OFF cells exhibit highly nonlinear excitatory integration^53,54^, when compared with those of the mouse retina. It is intriguing to think that more data, or tailored one-^18^ or two-dimensional^16^ stimuli, would allow such models to dissect more finely the distinct inputs integrated by different types of mammalian RGCs.

Naturally, the LNLN cascade only approximates the true nonlinearities present in RGC computation, and more detailed studies have been able to model particular elements of RGCs that cannot be captured in this general framework. Such nonlinear response properties include temporal precision^33,40^, nonlinear spatial integration^16,17,55,56^, contrast gain control^35,43^ and models based on mechanisms such as synaptic depression^57,58^ and presynaptic inhibition^45^. Such more detailed models will only be fittable in limited circumstances owing to the large number of component parameters and thus are not yet general. Likewise, the methods presented here are not ideal for characterization of direction-selectivity due to the non-separability of their RF components, which thus would require much larger amounts of data to adequately constrain and/or targeted visual stimuli. The problem of constraining more complex models is starting to be addressed using new machine-learning methods that can fit large populations of recorded neurons simultaneously^58,59^ and these provide an opportunity to fit large numbers of parameters with more data. Such techniques, however, are still in their infancy and currently have limited interpretability.

In this context, the sNIM is relatively simple: in a robust and user-friendly framework, it is able to approximate many of the nonlinearities revealed by more detailed studies. Assumptions about the specific structure of the model, such as space-time separability of the RFs, and the limit in the number of RF subunits, are necessarily approximations, but they maximize robustness and interpretability. Thus, the sNIM serves as a much-improved baseline model relative to the LN analysis that has dominated retinal computational analysis until recently.

### ON-OFF selectivity and the diversity of RGC computation

While making up only a small RGC population in cat and primate^59,60^, ON-OFF cells represent a significant fraction of the RGCs in mouse^13,61^. ON-OFF cells appear to be common in salamander retina as well^54,62^. Furthermore, an intriguing recent study utilizing LN analysis suggested that the response polarity of the linear receptive fields of mouse and pig RGCs varied with illumination intensity^63^: simple ON or OFF characterizations of retinal neurons therefore may be insufficient to capture their physiological functionality. Similar “polarity reversal” was observed in salamander OFF RGCs in response to “peripheral image shift”^64^, suggesting that robust methods to characterize ON-OFF responses are necessary^14^. The results we presented here demonstrate that the sNIM detects ON-OFF selectivity more sensitively than physiological characterizations using flashed stimuli and/or based on the linear RF^7,9,11^. The clear RFs detected also permit detailed characterization of the measured RFs (Fig. 1) and thus provide a framework for understanding the contribution of ON-OFF processing in vision.

More broadly, the revolution in mouse genetics has allowed for the discovery of new RGCs – e.g., J-RGCs^65^, and W3 cells^13^ – and the study of their presynaptic circuitry. Further, new anatomical and statistical techniques^5,6^ have amplified our understanding of novel and known RGC types. Nonlinear modeling approaches have offered a picture of cell diversity in more general stimulus contexts. The sNIM can be applied to general stimuli and offers a robust description of RGC computation that can be related to each cell’s stimulus selectivity, a diversity of nonlinear response properties, and potentially underlying mechanisms of processing within the visual circuitry.
